# Medical Professionals' Treatment Decisions for Critical Patients With Ambiguous Treatment Wishes: A Cross‐Sectional Study

**DOI:** 10.1002/hsr2.70564

**Published:** 2025-03-12

**Authors:** Kanako Yamamoto

**Affiliations:** ^1^ St. Luke's International University Tokyo Japan

**Keywords:** advanced care planning, decision aid, decision making, end‐of‐life, intensive care unit, shared decision making

## Abstract

**Background and Aims:**

Many patients do not want life‐prolonging treatments at critical stages of their illnesses, and are able to communicate their wishes to their families or surrogates. However, few among them have clarity on what such life‐prolonging treatments mean or entail. This study clarifies differences in the perspectives of medical professionals based on levels of expertise with respect to treatment decisions for intensive care unit (ICU) patients whose wishes for end‐of‐life care are ambiguous.

**Methods:**

The cross‐sectional study included intensivists, surgeons, and ICU nurses working in 171 Japanese hospitals with ICUs. A total of 837 participants were mailed questionnaires which included questions on the current status and approaches to decision‐making with end‐of‐life care for ICU patients. Additionally, a simulated case study was conducted to investigate the decision‐making process for treating patients with uncertain survival rates.

**Results:**

Responses were obtained from 489 participants from 151 hospitals. More than half of the intensivists and ICU nurses reported that family members' requests were respected when the patient had ambiguous treatment preferences. Participants considered the patients' survival probability when deciding to perform emergency surgery, with nurses having significantly higher thresholds than intensivists and surgeons (intensivists: 20.8 ± 20.6, surgeons: 26.6 ± 25.2, ICU nurses: 36.4 ± 26.4; *p* < 0.001).

**Conclusions:**

Family preferences take precedence over patient preferences when deciding on end‐of‐life treatments for ICU patients. Shared decision‐making should be promoted to correctly share patients' end‐of‐life medical wishes with surrogate decision‐makers and medical practitioners.

## Summary

- Life‐saving treatments may be considered in the intensive care unit (ICU), even if the patient advises their family of their desire not to receive life‐prolonging treatments.
- The methods and perceptions of decision support for patients and their families differ according to the expertise of the medical professionals, even for the same patient; this may lead to differences in setting treatment goals.
- In the ICU, where decision‐making time is short, medical professionals should make shared decisions while including families' information about patients' autonomy.

## 1 Introduction

Improving the quality of end‐of‐life care for intensive care unit (ICU) patients is a challenge [1]; therefore, efforts are being made to provide advance care planning (ACP) support [2, 3]. ACP is the process of discussing and sharing medical care plans with family members and healthcare professionals based on the patient's values [4]. However, the mechanism of ACP support and its effects on ICU patients remains unclear. One of the main functions of the ICU is to save lives and assist in the recovery of critically ill patients by providing multidisciplinary care. Therefore, the distinction between lifesaving and life‐sustaining treatments provided in an ICU is often unclear [5]. During end‐of‐life care for ICU patients, the decision and determination of treatment intensity vary from country to country [6]. These differences are problematic because they can create an imbalance between the quality of care received by patients and the accrued benefits [6].

Moreover, when considering treatment goals for critically ill patients, it is difficult for medical professionals to consider the patient's best interests [7, 8]. Medical professionals must decide whether the best goal of a patient's treatment is simply to aid survival or to maintain their quality of life after the treatment [9]. In recent years, there has been a tendency for patients to emphasize that the purpose of treatment is not limited to the preservation of life and that life should also be lived with dignity [10, 11]. A paucity of global standards exists with regard to the decision‐making process concerning end‐of‐life care in ICUs [6]. Thus, further research is necessary to elucidate the underlying reasons for this absence of standardization. For instance, in Japan, death with dignity is not legally recognized. Moreover, even in instances where a patient expresses a desire to have the ventilator removed, it is often challenging for medical personnel to proceed once the apparatus has been attached, thus giving rise to ethical dilemmas for hospitals and medical staff confronted with such circumstances.

## 2 Background

Many patients admitted to the ICU cannot make decisions or discuss their treatment goals owing to the effects of sedation or illness [7, 8]. Consequently, it is challenging to unequivocally substantiate the notion of “life‐prolonging treatment” for patients who were previously healthy [6, 7, 12]. This time is, therefore, characterized by the need for family members and medical professionals to make surrogate decisions on the patient's behalf regarding the goals of care [13]. It has been noted that deliberations among medical personnel can be arduous when formulating end‐of‐life care decisions for critically ill patients [14]. A salient factor that has been identified is the involvement of multiple physicians in the decision‐making process, which can result in divergent perspectives [15]. In many cases, multiple physicians participate in the patient's treatment, such as the Intensivist and the other department's attending physician; as a result, there is no single person in charge [16]. Additionally, discordances in opinion have been observed not only among physicians but also among ICU nurses [17]. Discrepancies in treatment policies among medical teams have also been documented [18]. Furthermore, there are instances where the treatment goals and care priorities of patients do not align between medical personnel [19]. Time limitations and a lack of knowledge are also factors [20]. The complexities involved in end‐of‐life care for patients in the ICU are manifold, and medical staff must navigate these challenges with care and diligence.

Furthermore, because ACP is not yet widely used [21], family members often fail to understand patients' wishes [2, 3]. Patients may convey to surrogate decision‐makers that they do not want life‐prolonging treatment; however, the specifics of these wishes may be ambiguous [9]. Physicians have little opportunity to confirm patients' treatment wishes with their families [22]. Thus, it is doubtful whether there is consistency between the life‐prolonging treatments being considered by medical professionals versus those being considered by family members. This ambiguity is a fundamental source of conflict and difficulty in life‐threatening situations for the ICU patients' surrogate decision‐makers. Hence, ICU patients may not receive their desired medical care, and their autonomy may not be respected by medical professionals. In clinical practice, it is recommended that the goals of patients at the terminal stage of illness should be discussed by a multidisciplinary team. However, the diverse values and judgments of medical professionals trying to agree on a single patient goal can lead to conflict.

Few studies have examined whether surrogate decision‐making differs depending on the expertise of the medical staff when the patient's definition of “life‐prolonging treatment” is vague. In the terminal stages of cancer, the assessment of the patient's remaining life expectancy is straightforward, and the concept of “life‐prolonging treatment” is readily comprehensible [9]. However, in settings such as the ICU or emergency departments, there is often a divergence in the understanding of the definition of “life‐prolonging treatment” among patients, their families, and medical staff [8, 9, 12]. In such cases, medical staff may tend to decide on the treatment policy for the patient based on their own sense of ethics, professional responsibility, and role [18, 23]. Furthermore, the process of determining the most suitable treatment for patients and the critical factors to be considered may vary depending on the type of occupation [24]. By identifying these factors, methods of supporting decision‐making may be developed that respect patient autonomy. Furthermore, the implementation of educational programs targeting medical staff on the topic of surrogate decision‐making and collaborative approaches holds promise in enhancing the quality of surrogate decision‐making. The resulting insights that could lead to the enhancement of end‐of‐life care for patients in the ICU. This study aims to clarify the characteristics and differences in medical professionals' perceptions of end‐of‐life treatment decision‐making for ICU patients with ambiguous wishes regarding life‐sustaining treatments.

## 3 Methods

### 3.1 Study Design, Participants and Definition

This cross‐sectional study was conducted in Japanese hospitals with ICUs. This study was part of a larger survey to develop a decision aid for ACP support for ICU patients [8]. The survey is a section on end‐of‐life care decision‐making in the ICU. Study participants included intensivists, surgeons, and ICU nurses from 400 randomly selected Japanese hospitals. Residents and nurses with two or fewer years of experience were excluded. An “ambiguous wish for life‐sustaining treatment” is defined as a patient telling a surrogate decision‐maker that no life‐sustaining treatment is desired, without providing additional detail. Thus, the patient's perceptions of life‐sustaining treatments and details of the desired treatment are unknown.

### 3.2 Sample Size Determination and Sampling Technique

This study used the analysis of variance (ANOVA). The correlation ratio estimation and power were set at 0.05 and 0.8, respectively. Consequently, the target sample size was set at 210 participants, with 70 participants in each profession. As this survey was originally conducted with ICU intensivists, surgeons, ICU nurses, ward nurses, and outpatient nurses, the sample size calculation differs from the planned number of participants for this survey. The survey hospitals were selected from among acute‐care hospitals in Japan that have ICUs, and the population was made up of 400 hospitals with ICU specialists on staff (random number table). The selection method used a cluster method to ensure that hospitals were selected from all regions of Japan. The response rate for postal surveys in Japan is estimated to be 30% [18, 25]. Therefore, it was assumed that approximately 30% (80‐120 hospitals) of the hospitals in the population would cooperate with the study. It was hypothesized that approximately 30% of the participants would, in turn, demonstrate cooperation with the facilities in question. It was determined that, of the hospitals that consented to participate in the survey, a sample of one to five physicians and nurses would be selected to participate. Next, it was anticipated that this would guarantee a minimum of 80 participants in each group, thereby ensuring sufficient statistical robustness.

### 3.3 Survey Period and Data Collection

The study period was from August to December 2019. Before starting the survey, 400 hospitals were contacted by mail, and 171 agreed to participate. Among intensivists, surgeons, and ICU nurses at these 171 hospitals, those who were available to cooperate with the survey were asked to participate, and the number of participants was used as the sample size. Ultimately, 155 intensivists, 127 surgeons, and 555 ICU nurses, for a total of 837 participants, completed the survey. The survey forms were sent to the hospital administrators, who selected the participants for the study. Participants received a description of the study (explaining the main purpose of the survey, survey method, and handling of personal information), the questionnaire, and a self‐addressed envelope. Consent was implicitly construed when the participants anonymously mailed the completed questionnaire using the self‐addressed envelope.

### 3.4 Survey Content

The main outcome measures were the decision‐making ability and judgment of ICU patients, as perceived by medical professionals; the status of treatment explanations provided to patients and family members; and the method of making decisions about end‐of‐life care. The answers were rated on a five‐point Likert scale. Additionally, two case studies were simulated, and in a free‐writing form, participants were asked to advise how they would respond to the wishes of the patients and their families. The case study details are provided in Supporting Information S1A. In Case 1, participants' response options were classified as (1) re‐operating, (2) not re‐operating, or (3) considering re‐operation. Participants were required to indicate an estimated survival probability rate (range 0%–100%) at which the lifesaving treatment should be withdrawn or withheld. In Case 2, the patient was placed on emergency ventilation, which was considered a lifesaving treatment with a high survival probability. Participant responses were analyzed in relation to the wishes of the surrogate decision‐makers following an explanation of the treatment; the responses were: (1) intubate, (2) do not intubate, or (3) consider intubating. Furthermore, the participants answered multiple questions about what explanation they would provide surrogate decision‐makers before initiating treatment. The other survey parameters included age, sex, work experience, and position. All questionnaires were original. This survey was developed through discussions between researchers who are familiar with end‐of‐life care, nursing, and decision‐making support at the ICU. The survey was then shown to physicians and nurses working at the ICU to check for any unclear expressions. Through these processes, the appropriateness of the survey content was confirmed.

### 3.5 Statistical Analyses

Data were analyzed using IBM SPSS Statistics for Windows (version 25.0; IBM Corp., Armonk, NY, USA). The data were tested for normality and were descriptively analyzed. The differences between the three participant occupations were examined using a one‐way ANOVA. A *p*‐value < 0.05 was considered significant. The free‐text responses in the case were categorized by semantic content using content analysis techniques. Tukey's multiple comparison test was performed whenever significant differences of less than 5% were found. In instances where equal group variance could not be assumed, Welch's test was employed, and when a significant difference at < 5% was observed, Game‐Howell's multiple comparisons were performed. The *t*‐test was conducted to analyze two groups with different occupations and departments. Finally, the *χ* ^2^ test was performed for the nominal scale, and the significance level was set as < 5% (two‐sided test). All statistical analyses were carried out under the supervision of trained statisticians.

### 3.6 Ethical Considerations

The Research Ethics Committee of the author's institution approved this study (approval number: 19A036). The participants were informed about the purpose of the study, the survey method, the voluntary nature of participation in the study, protection of personal information, disclosure of results, and withdrawal of consent. The data were coded and anonymized, and are therefore not personally identifiable.

## 4 Results

A total of 366 individuals (response rate: 43.7%) responded to the questionnaire, including 124 intensivists, 59 surgeons, and 183 ICU nurses. Table 1 summarizes the participants' characteristics.

**Table 1:** Summary of participants.

	Intensivist		Surgeon		ICU nurse	
	*n*	%	*n*	%	*n*	%
Age, mean ± SD	45.7 ± 10.0		46.3 ± 9.4		35.3 ± 7.3	
Years of work experience, mean ± SD	20.3 ± 9.2		21.0 ± 9.5		12.6 ± 6.9	
Position						
Manager	38	30.6	17	28.8	14	7.7
Chief	28	22.6	20	33.9	29	15.8
Staff	58	46.8	22	37.3	140	76.5
Clinical department						
Gastroenterology			22	37.3		
Cardiovascular			23	39		
Thoracic surgery			3	5.1		
Neurosurgery			10	16.9		
Other			1	1.7		
Education						
Junior college or technical school					136	74.3
College	96	77.4	38	64.4	46	25.1
Graduate school	28	22.6	21	35.6	4	2.2
Type of ICU						
Closed‐ICU	28	22.6	3	5.1	21	11.5
Semi‐closed	79	63.7	42	71.2	89	48.6
Open‐ICU	17	13.7	14	23.7	73	39.9

*Note:* Total numbers of intensivists, *n* = 124; surgeons, *n* = 59; ICU nurses, *n* = 183. A closed ICU is a facility where only intensivists make decisions on all the treatment strategies for patients. In semi‐closed ICUs, intensivists intervene for all patients in the ICU or those in need. An ICU without an intensivist is an open ICU.

Abbreviation: SD, standard deviation.

### 4.1 End‐of‐Life Care in the ICU

Forty‐one intensivists, 19 surgeons, and 30 ICU nurses responded that end‐of‐life care was often provided based on the understanding that the patients themselves wanted to be treated (*p* < 0.001) (Table 2). Additionally, 57 intensivists, 62 ICU nurses, and 32 surgeons reported that they would prioritize patients' treatment intentions over those of surrogate decision‐makers (*p* < 0.001).

**Table 2:** Medical professionals' perceptions of end‐of‐life care for patients in ICUs.

	Intensivist		Surgery		ICU Nurse		*p* *
	*n*	%	*n*	%	*n*	%	
Can it be considered along with the patient's wishes or needs?							0.006
Never	7	5.7	8	13.6	15	8.2	
Occasionally	39	34.9	24	40.7	74	40.4	
Sometimes	36	29.2	15	25.4	64	35.0	
Often	33	22.6	8	13.6	25	13.7	
Always	8	7.5	4	6.8	5	2.7	
Conducting a multidisciplinary conference							< 0.001
Never	1	0.9	9	15.3	13	7.1	
Occasionally	28	25.5	26	44.1	42	23.0	
Sometimes	43	36.8	12	20.3	74	40.4	
Often	41	32.1	7	11.9	46	25.1	
Always	11	4.7	5	5.9	7	3.8	
The degree to which you speak at a conference							< 0.001
Never	5	4.1	6	10.5	16	8.7	
Occasionally	16	13.0	19	33.3	53	29.0	
Sometimes	29	23.6	12	21.1	73	39.9	
Often	48	39.0	12	21.1	33	18.0	
Always	25	23.0	8	14.0	7	3.8	
Situations where the patient and family have different opinions for treatment							< 0.001
Never	2	1.6	8	14.0	7	3.8	
Occasionally	45	36.9	25	43.9	57	31.1	
Sometimes	48	39.3	22	38.6	76	41.5	
Often	27	22.1	2	3.5	38	20.8	
Always	0	0	0	0	3	1.6	
Which is more likely to be the patient's or family's wish?							
Patient	57	44.7	32	58.2	62	33.9	
Family	63	55.3	23	41.8	114	62.3	
Whether there are conflicts between the medical professional and patients' family or surrogates							0.002
Never	16	13.3	21	36.8	42	23.0	
Occasionally	76	62.9	31	54.4	91	49.7	
Sometimes	24	19.0	4	7.0	40	21.9	
Often	6	4.8	1	1.8	9	4.9	
Always	0		0		0	0.0	
The mental burden of considering end‐of‐life care for patients							0.216
Never	5	4.7	3	5.3	8	4.4	
Occasionally	45	36.8	21	36.8	40	21.9	
Sometimes	32	27.4	14	24.6	63	34.4	
Often	30	23.6	12	21.1	60	32.8	
Always	9	7.5	7	12.3	11	6.0	

*Note:* Total numbers: intensivists, *n* = 124; surgeons, *n* = 59; ICU nurses, *n* = 183.

* One‐way ANOVA.

### 4.2 Patient Decision‐Making Capacity and Patients' and Family Members' Understanding During ICU Admission

Medical professionals' views on the extent to which patients or surrogate decision‐makers understood treatment‐related explanations of ventilator support were examined (Table 3). The probability rates (range 0–100) of participants' perceptions of patients' understanding of their explanations were 43.0 ± 19.5, 41.1 ± 20.7, and 60.1 ± 20.0 for intensivists, surgeons, and ICU nurses, respectively (*p* < 0.001). Moreover, participants perceived surrogate decision‐makers' understanding of the explanations (range 0–100) to be 67.9 ± 15.3, 72.3 ± 18.3, and 72.5 ± 15.6 for intensivists, surgeons, and ICU nurses, respectively (*p* = 0.040).

**Table 3:** Medical professionals' perceptions of decision‐making capacity and understanding of patients in ICUs.

	Intensivist		Surgeon		ICU Nurse		*p* *
	*n*	%	*n*	%	*n*	%	
Provide explanations to patients and understand them							
Without a ventilator (common or usual)	63	50.8	48	81.4	168	91.9	< 0.001
Degree of patients' understanding (VAS), mean ± SD	59.4 ± 18.2		61.7 ± 19.1		69.3 ± 17.0		< 0.001
With a ventilator (common or usual)	42	33.9	20	33.9	167	91.3	< 0.001
Degree of patients' understanding (VAS), mean ± SD	43.0 ± 19.5		41.1 ± 20.7		60.1 ± 20.0		< 0.001
Criteria for judging decision‐making ability							
Agitation‐sedation scale	84	68.3	18	30.5	144	78.7	
Not delirium	110	92.3	49	83.1	148	80.9	
No use of continuous analgesics	11	9.6	11	18.6	26	14.2	
No use of continuous sedatives	43	34.7	27	45.8	61	33.3	
Consciousness level	55	44.4	15	42.4	68	61.7	
No previous dementia	60	48.4	27	45.8	94	51.4	
Other	11	8.9	3	5.1	11	6.0	
Difficulty in judging a patient's decision‐making ability (sometimes ‐ always)	108	87.1	41	69.5	96	52.5	< 0.001
Explanation and understanding of the patient's family and surrogate decision‐makers							< 0.001
Providing explanations to family members or surrogate decision‐makers							
Never	4	1.9	1	1.7	3	1.6	
Occasionally	35	30.2	9	15.3	9	4.9	
Sometimes	36	30.2	6	10.2	30	16.4	
Often	32	23.6	23	39.0	93	50.8	
Always	17	14.2	20	33.9	48	26.2	
Degree of families or surrogate decision‐maker's understanding (VAS) mean ± SD	67.9 ± 15.3		72.2 ± 18.3		72.5 ± 15.6		.004

Abbreviations: SD, standard deviation; VAS, visual analog scale.

* One‐way ANOVA.

### 4.3 Treatment Wishes of ICU Patients

**Figure 1:**
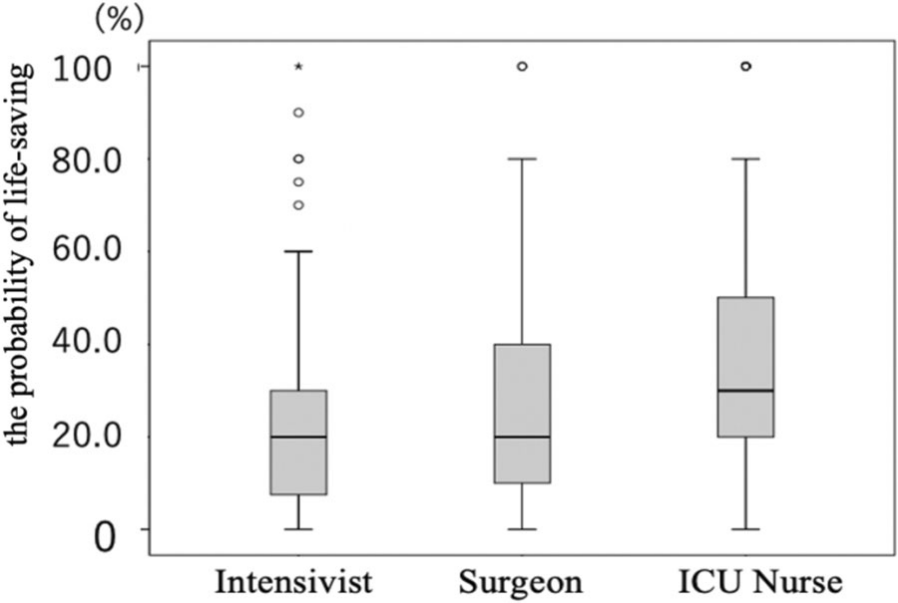
Medical professionals' perception of lifesaving probability to consider withholding/withdrawal for patients with ambiguous treatment wishes.

Eighteen intensivists and 10 surgeons responded that they would not re‐operate (Table 4). Surgeons were more likely than intensivists to respond that, owing to perioperative complications, a re‐operation was lifesaving rather than life‐sustaining. By contrast, intensivists were more likely to try to awaken the patients and make decisions for them whenever possible. The specific responses of 160 ICU nurses (Table 5) included 94 supporting family members, 41 coordinating and collaborating with physicians, and 13 confirming the patients' intentions. Furthermore, in this patient's case, the survival probability rate for withholding or providing treatment differed according to specialty: 20.8 ± 20.6% (mean ± standard deviation), 26.6 ± 25.2%, and 36.4 ± 26.4% for intensivists, surgeons, and ICU nurses, respectively (intensivists vs. surgeons, *p* = 0.148; intensivists vs. ICU nurses, *p* < 0.001; surgeon vs. ICU nurse, *p* = 0.021; Figure 1). Nineteen intensivists (15.3%), 9 surgeons (15.3%), and 19 ICU nurses (10.0%) responded that they would not withhold lifesaving treatment, even if the survival probability decreased to 0%–5%, and the patient did not intend to seek life‐sustaining treatment. All physicians indicated their intention to continue lifesaving treatment until the patient's survival rates decreased to an extremely low level (*p* < 0.001). However, ICU nurses were less likely to consider re‐operation unless the survival rate was as high as 90%–100% (*p* < 0.001).

**Table 4:** Perception of intensivists and surgeons of the cases.

	Intensivist		Surgeon	
	*n*	%	*n*	%
*Case 1*				
Recommend surgery	38	30.6	21	35.6
Consider	68	54.8	28	47.5
Discuss within the department	11	8.9	4	6.8
Multidisciplinary conference	16	12.9	5	8.5
Confirm with the patient	13	10.5	6	10.2
Discussion between family members and healthcare providers	36	29.0	27	45.8
Clinical ethics conference	5	4.0	2	3.4
Others	3	2.4	1	1.7
No surgery	18	14.5	10	16.9
*Case 2*				
Recommend intubation	26	21.0	11	18.6
Consider	85	68.5	44	74.6
Discuss within the department	11	8.9	23	39.0
Multidisciplinary conference	17	13.7	4	6.8
Confirm with the patient	33	26.6	6	10.2
Discussion between family members and healthcare providers	77	62.1	33	55.9
Clinical ethics conference	7	5.6	4	6.8
Consideration of other respiratory support devices	23	18.5	2	3.4
Collaboration with the palliative care team	11	8.9	2	3.4
No intubation	13	10.5	4	6.8

*Note:* Total numbers: intensivists, *n* = 124; surgeons, *n* = 59.

**Table 5:** ICU nurses' perceptions of the cases.

	ICU nurse	
	*n*	%
*Case 1*		
Take action	160	87.4
Approaches to physicians	41	25.6
Approach to the patient's family	94	58.8
Holding and adjusting multidisciplinary conferences	15	9.4
Approach to patients	13	8.1
Discussion between nurses	5	3.1
Others	5	3.1
Do not take action	23	12.6
*Case 2*		
Take action	142	77.6
Approaches to physicians	55	34.4
Approach to the patient's family	82	51.3
Holding and adjusting multidisciplinary conferences	14	8.8
Approach to patients	5	3.1
Discussion between nurses	28	17.5
Do not do action	41	22.4

*Note:* Total number of ICU nurses, *n* = 183. Details of “Take action” have duplicate answers.

In addition, Case 2 depicted a “decision on whether to re‐intubate and place the patient on ventilatory support due to postoperative complications.” In Case 2, 13 intensivists and 4 surgeons indicated that reintubation would not be performed (Table 4). A characteristic of intensivists was that many answered that they would contact the patient. Surgeons, on the other hand, were more likely to discuss the matter within the department.

## 5 Discussion

This survey aimed to clarify the differences in and characteristics of end‐of‐life treatment perceptions among ICU patients with ambiguous wishes regarding life‐prolonging treatment from the perspectives of three different professions. Intensivists and ICU nurses felt that it was difficult to confirm patients' treatment wishes, and they recognized differences in opinion between patients and their families as well as understood that there may be conflicts with families. Previous studies have also reported that there is a tendency for conflicts to occur between intensivists and ICU nurses and surrogate decision‐makers when considering treatment policies for patients who lack the ability to make decisions [22, 26]. In making treatment decisions for ICU patients, even when there is less hope of recovery, there is still hope for the patient's life; even when there is hope for recovery, there is often a high possibility of a sudden change, so it is easy for such situations to become complicated [12]. In this survey, too, there were differences in end‐of‐life care decisions between intensivists and surgeons, and it was inferred that the existence of multiple treatment managers made decision‐making difficult. When making complex and difficult decisions, it is often the case that we rely on guidelines. However, guidelines for end‐of‐life care and palliative care in the ICU have few indicators with clear evidence [27, 28]. The basic policy recommends that the best interests of the patient be discussed and decided upon by a multidisciplinary team. The differences in the expertise and responsibilities of healthcare professionals may naturally lead to differences in thinking about what is best for the patient. Even among intensivists, surgeons tended to be more likely to choose aggressive treatment with a view to the possibility of saving the patient's life when compared to intensivists. This difference may cause conflict between medical staff at the ICU [29, 30]. Each medical staff must respect each other's responsibilities and roles, to try to understand each other's suffering, and to work together [31, 32]. Furthermore, compared to physicians, nurses demonstrated higher ratings to the understanding of treatment explanations and the decision‐making ability of patients with ventilators. The fact that nurses spend more time with patients than physicians may have had an effect. When deciding on a patient's treatment goals, it is also important for medical staff to share their thought processes and communicate fully with each other [18, 33]. Patients receiving treatment in an acute care hospital are rarely treated by a single physician. Patients interact with medical staff in various departments, including outpatient clinics, hospital wards, and ICUs. It may be necessary to devise a system‐level approach to ensure that patients' end‐of‐life wishes are clearly and seamlessly passed on.

In addition, when considering the goals of end‐of‐life care for ICU patients, it was found that the wishes of the family are likely to be incorporated and that it is still difficult to respect the full autonomy of the patient. The reasons for this can be inferred from the results of this study, which show that it is often not possible to confirm the wishes of the patient and that there are often differences in wishes between the patient and the family. There are differences between the treatment goals of surrogate decision‐makers and the treatment those of patients [30, 31]. To practice higher quality end‐of‐life care for patients, it has been suggested that medical professionals should improve ethical practice and that clear intentions should be expressed through ACP and AD [32]. However, it is difficult to determine how far ACP should be applied in treatment situations in the ICU, where there is a possibility of saving lives. In Japan, there are reports that even patients who had expressed their intention to receive DNAR were likely to receive resuscitation treatment during emergency transport [34]. In addition to encouraging ICU patients and their families to prepare ACP, medical staff need to fully explain the benefits and risks of the treatment to be received by the patient and to provide support in improving the quality of decision‐making. It is important to not only confirm the DNAR intention with the patient, but also to share the process by which that conclusion was reached. Furthermore, it is important to concretize what life‐prolonging treatment means for the patient and share that with both the medical staff and the patient and family. To make such high‐quality decisions, medical staff need to provide sufficient information, including the advantages and disadvantages of the treatment options [35]. In recent years, there has been an increasing demand for healthcare professionals to understand and to be able to implement communication techniques and decision‐making support methods for shared decision‐making (SDM) [36]. When making treatment decisions at the end of life, it is important to check that patients and their families are able to understand and recognize the situation correctly, and to support them in promoting SDM so that they can make decisions based on the patient's values. The effects of SDM on ICU patients are still unclear, and SDM's implementaion is difficult. In addition, the outcomes of SDM approaches and support for surrogate decision‐makers regarding end‐of‐life medical decisions are also still unclear. Treatment options and decisions in the ICU are often time‐sensitive and difficult for patients to understand due to the many medical issues involved. In addition, it is often difficult to say that a patient will definitely recover as a result of treatment. For this reason, it is thought that respecting patient independence would better arise from devising ways to share treatment options with patients and their families while paying attention to the patient's “values” before then making joint decisions. It may be necessary for medical staff to improve their education regarding SDM and to evaluate their practice [37]. Future research is needed to evaluate the effectiveness of healthcare professionals' SDM support and build evidence. This is expected to lead to improvements in the quality of decision‐making and end‐of‐life care for ICU patients.

This study has some limitations. First, there was a slight bias in the participation of the target surgeons. However, efforts were made to increase the response rate as much as possible by sending letters to the facilities where the participants were affiliated. Although the specialties of the surgeons involved in the treatment of ICU patients varied, it was difficult to include participants from all specialties comprehensively. Second, the sample population included physicians and nurses in Japan, which limited the generalizability of the results. Finally, although the participants' opinions were evaluated using two case studies, these simulations offered limited information and response choices.

## 6 Conclusion

This study found that in scenarios where patients do not clearly state whether to withdraw or continue life‐sustaining treatments, then their families' wishes would tend to take precedence. Medical professionals also make judgments regarding lifesaving treatments. Importantly, multidisciplinary discussions should be held with the various medical professionals involved in patient care, along with surrogate decision‐makers, to accurately evaluate patients' treatment intentions. To improve the quality of end‐of‐life care for ICU patients, medical professionals should help patients and surrogate decision‐makers make satisfactory and acceptable decisions. Hence, medical professionals caring for ICU patients must improve their SDM skills.

## Author Contributions

**Kanako Yamamoto:** conceptualization, data curation, formal analysis, funding acquisition, investigation, methodology, project administration, writing – original draft, writing – review and editing.

## Ethics Statement

This study was approved by the Research Ethics Committee of St. Luke's International University (approval number: 19‐A036).

## Consent

This study did not require the participation of patients.

## Conflicts of Interest

The author declares no conflicts of interest.

## Transparency Statement

The lead author Kanako Yamamoto affirms that this manuscript is an honest, accurate, and transparent account of the study being reported; that no important aspects of the study have been omitted; and that any discrepancies from the study as planned (and, if relevant, registered) have been explained.

## Supporting information

Supplementary Information

## Data Availability

The data sets used and/or analyzed during the current study are available from the corresponding author on reasonable request.
