# Supplementary material for: Medical Professionals' Treatment Decisions for Critical Patients With Ambiguous Treatment Wishes: A Cross‐Sectional Study
Source: Health Sci Rep. 2025 Mar 12;8(3):e70564. doi: 10.1002/hsr2.70564 (PMC11903493; doi:10.1002/hsr2.70564)
Supplement: Supplementary file 1 — Supplementary Information [file HSR2-8-e70564-s001.docx]

**Additional file A**

The cases study were as follows.

Case1

Patient A, a 75-year-old man, was admitted to the ICU for a scheduled surgery, but developed postoperative complications. Ventilator support was provided to him, along with analgesic and sedative medications. Owing to his condition, he was deemed incapable of making decisions. Before re-operating, the physician informed the family members that, if re-operation was not performed, the patient had a minimal chance of survival, estimated at 50%. Without the re-operation, the patient could die within a few days. The family members were aware that the patient had refused life-sustaining treatment. Therefore, it was difficult to decide whether surgery should be performed.

**Case 2**

Patient B was an 80-year-old woman. Following surgery, she was assessed for temporary intubation because of aspiration pneumonia and was admitted to the ICU. She had a survival probability of 80%. The physician explained the situation to the family. The family strongly opposed the use of mechanical ventilation and stated that “the patient did not wish to be placed on a ventilator.” The patient experienced significant respiratory distress and had insufficient decision-making capacity.
